# A Comparison of Traditional Chinese Medicine and Multiple Conventional Therapy in Treating Oral Lichen Planus: A Network Meta-analysis

**DOI:** 10.3290/j.ohpd.b5779166

**Published:** 2024-10-14

**Authors:** Hoilun Chu, Yanting Ip, Guilin Yang

**Affiliations:** a Undergraduate Student in Stomatology, School of Stomatology, Jinan University, China. Methodology, investigation, data curation, writing (original draft, visualisation).; b Master’s Student in Nutrition and Health, School of Stomatology, Jinan University, China. Conceptualisation, methodology, supervision, writing (review and editing), project administration, funding acquisition.; c Undergraduate Student in Stomatology, School of Stomatology, Jinan University, China. Software, investigation, formal analysis, writing (original draft).

**Keywords:** efficacy analysis, network meta-analysis, oral lichen planus, photodynamic therapy, traditional Chinese medicine

## Abstract

**Purpose:**

To evaluate and compare the efficacy of seven conventional treatments and traditional Chinese medicine (TCM) combined therapies for oral lichen planus.

**Materials and Methods:**

This study employs PubMed, Web of Science, Cochrane Library, and Cnki to collect studies. After evaluating the quality and bias risk, RevMan 5.4.1 and R Gemtc package was utilised with a visual analogue scale and side effects as outcomes, to compare the efficacy of the seven treatments.

**Results:**

This study included 20 studies, with a sample size of 1669. Our results suggest that photodynamic therapy and TCM demonstrate the most significant decrease in visual analogue scale and the rank is as follows: photodynamic therapy > TCM > TCM combined with non-hormonal immunosuppressive drugs > TCM combined with glucocorticoids > chloroquine combined with glucocorticoids > non-hormonal immunosuppressive drugs > glucocorticoids. Among them, compared to glucocorticoids, photodynamic therapy (–1.55, 95% CI: (–3.09, –0.02)), TCM (–1.25, 95% CI: (–2.46, –0.06)) significantly outperform in statistics. Moreover, no side effects were reported by the photodynamic therapy treatment. In the comparison with non-hormonal immunosuppressive drugs, the result indicates TCM (–4.17, 95% CI (–8.24, –0.34)), glucocorticoids (–2.78, 95% CI (–5.69, –0.17)) and their combination (–2.83, 95% CI (–5.93, –0.05)) have a significantly lower probability of the appearance of side effects.

**Conclusion:**

This study indicates that TCM, from the perspectives of efficacy and the likelihood of side effects, outperforms all other common therapies, besides photodynamic therapy, in treating oral lichen planus.

Oral lichen planus (OLP), first described by Wilson in 1869, is a mucocutaneous disease affecting the oral mucosa. Clinically, OLP is categorised into erosive and non-erosive types. Its common symptoms include white striae, white papules, white plaques, erythema, erosion, or vesicles, and principal areas of damage typically include the buccal mucosa, tongue, and gums. According to the study, the prevalence of OLP ranges from 0.1% to 4.0% and the incidence is approximately 1.27%, ranking second among oral whole mucosal diseases.8 OLP generally appears in individuals in the age range of 30 to 60, especially females.^22^ In the academy, OLP is regarded as a potential aura of cancer.^7^ Research suggests that dysregulation and mutations in apoptosis and cell proliferation may lead to the occurrence of malignant transformation in OLP. Therefore, OLP presents a certain tendency towards carcinogenesis, being able to transform into oral squamous cell carcinoma (OSCC).^36^

Since the mechanism of OLP pathogenesis and carcinogenesis is still unclear, there is no specific treatment for OLP in clinical practice. However, it is widely believed in academia that the pathogenesis of OLP may involve a T-cell-mediated autoimmune response facilitated by Langerhans cells (LC) as antigen-presenting cells. Therefore, the treatment of glucocorticoids (GC) is often the first choice in clinical.^20^ It would be a long-term treatment for OLP and patients are required to keep taking the GC for extended periods to alleviate symptoms. However, a series of side effects, such as bacterial infections, weakened immune systems, hypertension, and hyperglycaemia, are accompanied by the long-term use of GC.^6^

Some patients with OLP are not suitable for using GC as a treatment due to various reasons such as pregnancy, hypertension, diabetes, etc. Therefore, many non-hormonal alternative treatments have been developed. Commonly used alternatives include photodynamic therapy (PDT), non-hormonal immunosuppressive drugs (ID), chloroquine combined with glucocorticoids (CQ+GC), etc. In recent years, traditional Chinese medicine (TCM) therapies have been widely accepted by clinicians, since they can offer the following advantages. First, TCM has its therapeutic theories and long-term clinical experience, which can explain diseases with unclear pathogenesis, such as OLP, and provide corresponding herbal treatments. Second, compared to Western medicine, TCM treatments are relatively more economically efficient, reducing the financial burden on patients and society. In the past, researchers engaged the paired comparison among the treatments, but the comprehensive evaluations among various treatment combinations are limited. This study utilises a network meta-analysis (NMA) to analyse the clinical efficacy studies published from January 2013 to December 2023. We aim to compare the efficacy of different treatments and their combinations, thus determining the optimal therapy from the perspectives of efficacy and side effects, and providing treatment advice in clinical cases. This study has been registered in PROSPERO with registration number CRD42024523176.

## MATERIALS AND METHODS

### Inclusion Criteria

In this research, we selected studies according to the following criteria:

1. The language of the study literature is either Chinese or English.
2. The study design is either a prospective cohort study or a randomised controlled trial (RCT) study.
3. Study samples must all be diagnosed as patients with OLP and have no diagnosed other oral diseases.
4. The comprehensive efficacy evaluation of the study needs to include pre-treatment and post-treatment visual analogue scale (VAS) scores, which are demonstrated with standardised mean difference (SMD).
5. For RCT studies, the treatment measures in the Observation group are one of the following: GC, ID, TCM, TCM combined with GC (TCM+GC), TCM combined with ID (TCM+ID), CQ+GC, and PDT. The treatment measures in the control group must also be one of them, excluding the intervention groups.

### Exclusion Criteria

1. There is no mention that patients in the study are excluded from other oral diseases but OLP.
2. Research outcome measures included VAS scores but lacked variables such as SD.
3. Any treatment excluding GC, ID, TCM, TCM+GC, TCM+ID, CQ+GC, and PDT is involved in the study.
4. Literature types included reviews, systematic reviews, experience summaries, conference papers, and theses.
5. Literature was in languages other than Chinese and English.

### Search Strategy

A thorough search of various databases including PubMed, Web of Science, Cochrane Library, and Cnki was conducted. The terms ‘Kouqiangbianpingtaixian’ and ‘Liaoxiao’ were searched in the Chinese database and the terms ‘OLP’, ‘treatment’ and ‘efficacy’ were searched in the English database. All publications from 2013 to 2023 were collected, then entered into the pre-inclusion procedures, and checked by two reviewers based on inclusion and exclusion criteria.

### Selection Process

Two reviewers independently conducted searches in databases using a predefined search strategy. They screened studies based on titles and abstracts against inclusion criteria, excluding irrelevant and ineligible ones. Then, they independently reviewed the full-text articles of the selected studies, excluding those that met the exclusion criteria. In cases of discrepancies, a third reviewer made the final decision on study inclusion. Following screening, the two reviewers independently extracted data and entered them into an Excel spreadsheet. If there were discrepancies in data extraction, a third reviewer performed data extraction again. Ultimately, 20 relevant studies were included, with a total sample size of 1669. Extracted data included author names, publication years, randomisation methods, sample sizes, intervention measures, control group treatments, number of outcome indicators, adverse reactions, and so forth.

### Risk of Bias Assessment

The process of assessing the risk of bias in included studies involved two reviewers working independently. Before this, both reviewers underwent identical bias risk assessment training and successfully passed a risk assessment test administered by professionals. Throughout the assessment, strict adherence to the Cochrane Handbook’s guidelines for evaluating bias risk in RCTs was maintained. Each study included was evaluated using the RCT bias risk assessment tool within RevMan 5.4.1, covering aspects such as allocation concealment, random sequence generation, blinding procedures for participants, intervention providers, and outcome assessors, completeness of outcome data, selective reporting, and other potential biases. Evaluation reports were documented in an Excel spreadsheet. In instances of conflicting results, the two reviewers engaged in discussion; if consensus remained elusive after discussion, a third researcher was brought in to conduct the assessment.

### Statistical Analysis

Following the instruction of Prisma NMA guidelines, the comprehensive evaluation of efficacy in this study is based on utilising odds ratios (ORs) for dichotomous outcomes and SMD for continuous outcomes through NMA, which was conducted using the Rajas package for seven interventions, employing four Markov chains with 50,000 iterations and 20,000 annealing cycles for consistency. Inconsistency tests and consistency models were utilised to assess study consistency. The GEMTC package in R software was utilised to generate probability ranking graphs, calculate the surface under the cumulative ranking curve (SUCRA) values, and determine mean ranks for the interventions. Higher SUCRA values, which means closer to 1, indicate better treatment effects and higher rankings, while lower values, which means closer to 0, suggest poorer treatment effects and lower rankings. In the bar chart probability plot, higher probability values indicate a higher likelihood of the intervention being ranked. Furthermore, a funnel plot was created using RevMan 5.4.1 software to identify potential publication bias.

## RESULTS

### Literature Search

**Fig 1:**
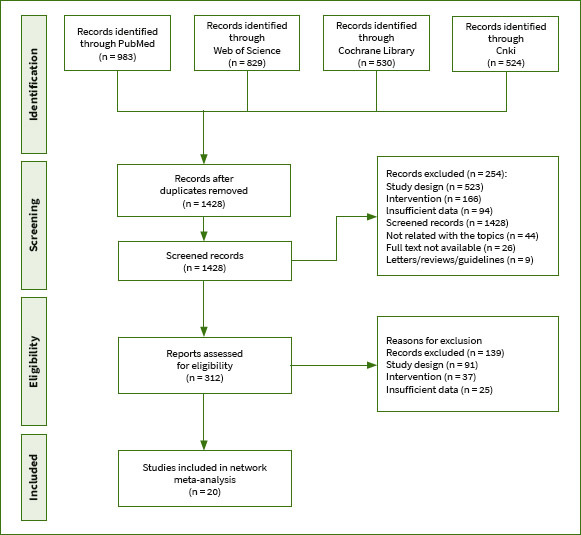
Flow diagram.

We conducted a comprehensive search and identified a total of 2866 relevant articles across various databases, including PubMed (n = 983), Web of Science (n = 829), Cochrane Library (n = 530), and CNKI (n = 524). After removing duplicates, we were left with 1428 articles. Initial screening based on titles and abstracts led to the exclusion of 1116 articles. Subsequently, we thoroughly reviewed the full texts of the remaining 312 articles. Finally, after this detailed evaluation, we selected 20 articles that met our predefined inclusion criteria. The detailed procedure for literature inclusion is delineated in Figure 1.

### Study Characteristics and Risk of Bias

**Fig 2:**
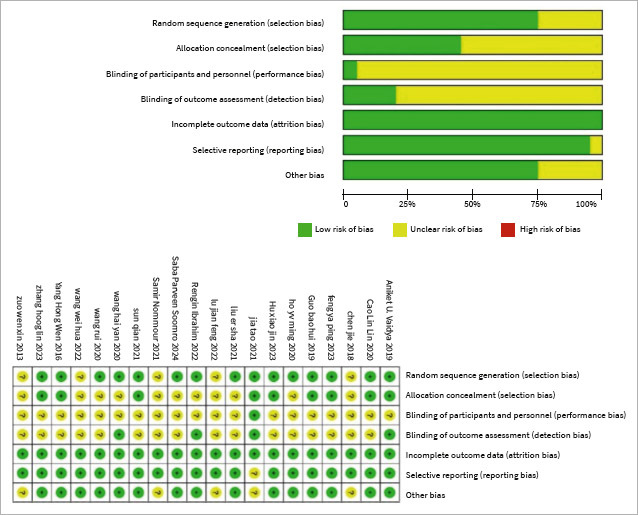
Risk of bias.

The analysis incorporated 20 studies, involving a total of 1669 samples and encompassing seven different treatments: TCM, TCM+ID, TCM+GC, CQ+GC, ID, GC, and PDT. Among studies, 8 of them outlined the doing of randomisation methods, while the remaining did not provide such details. The duration of treatment varied from 1 week to 24 weeks, with most studies falling within the 4–12-week range. Outcome measures primarily focused on VAS scores and the occurrence of side effects. Notably, 10 studies documented a combined total of 97 side effect cases. For a more comprehensive understanding of the studies, detailed information can be referenced in Table 1, while the results of the risk of bias assessment are visually represented in Figure 2.

**Table 1:** Baseline data of the included studies

Study	Random method	Gender		Average age		Treatment		Frequency	Outcomes	Side effect
		Male	Female	Observation group	Control group	Observation group	Control group			
Aniket et al^3^	N/A	19	41	N/A		TCM+GC	GC	8 weeks	①	N/A
Lingling et al^21^	Random number table	52	44	47.54 ± 10.30	48.01 ± 9.98	TCM+GC	GC	12 weeks	① ②	10
Jie et alet al^19^	N/A	46	79	49.93 ± 9.29	49.84 ± 9.37	TCM+GC	GC	24 weeks	① ②	19
Yaping et al^37^	Random number table	40	48	41.38 ± 12.11	41.32 ± 12.06	TCM+GC	GC	4 weeks	① ②	7
Baohui et al^4^	Random number table	36	44	48.90 ± 9.50	49.10 ± 10.40	TCM+GC	GC	4 weeks	①	N/A
Yuming et al^38^	N/A	34	56	41.19 ± 6.83	41.30 ± 6.74	TCM+GC	GC	12 weeks	①	N/A
Xiaojing et al^35^	Random number table	31	49	54.67 ± 5.76	55.03 ± 5.81	TCM+GC	GC	12 weeks	①	N/A
Tao et al^32^	Random number table	57	27	41.73 ± 5.84	41.85 ± 5.80	ID	GC	8 weeks	①	N/A
Ersha et al^10^	N/A	49	41	46.21 ± 5.56	45.72 ± 5.14	CQ+GC	GC	1 weeks	① ②	5
Jianfeng et al^18^	N/A	27	45	37.14 ± 5.97	36.75 ± 5.72	TCM+ID	ID	12 weeks	①	N/A
Rengin et al^26^	N/A	NA		N/A		PDT	GC	9 weeks	①	N/A
Saba et al^28^	N/A	22	38	N/A		ID	GC	4 weeks	①	N/A
Samir et al^31^	N/A	32	64	48		PDT	GC	6 weeks	①	N/A
Qian et al^25^	Random number table	49	55	45.28 ± 7.33	45.31 ± 7.26	TCM+GC	GC	3 weeks	① ②	11
Haiyan et al^13^	N/A	34	46	35.50 ± 6.70	34.80 ± 5.60	TCM+ID	ID	12 weeks	① ②	2
Rui et al^27^	N/A	43	39	49.57 ± 7.86	50.04 ± 7.91	TCM+GC	GC	12 weeks	①	N/A
Weihua et al^33^	N/A	47	37	42.46 ± 8.74	40,90 ± 9.23	TCM	GC	12 weeks	① ②	8
Hongwen et al^16^	Random number table	43	57	47.10 ± 5.20	47.00 ± 5.20	TCM+GC	GC	12 weeks	① ②	7
Hongling et al^15^	Random number table	43	67	50.33 ± 5.49	49.15 ± 6.28	TCM+GC	GC	8 weeks	① ②	13
Wenxin et al^34^	N/A	27	35	49.52 ± 13.64	50.45 ± 13.72	ID	GC	12 weeks	① ②	17

Note: ① VAS Score ② Side effect N/A = Not mentioned; GC: glucocorticoids; PDT: photodynamic therapy; TCM: traditional Chinese medicine, ID: non-hormonal immunosuppressive drugs; CQ+GC: chloroquine combined with glucocorticoids; TCM+GC: traditional Chinese medicine combined with glucocorticoids; TCM+ID: traditional Chinese medicine combined with non-hormonal immunosuppressive drugs.

**Fig 3:**
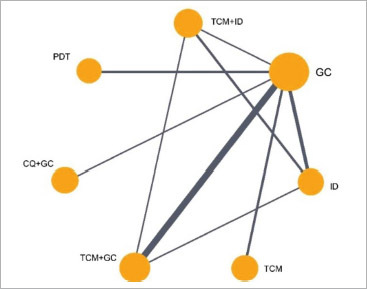
Network evidence plot for VAS scores. GC: glucocorticoids; PDT: photodynamic therapy; TCM: traditional Chinese medicine, ID: non-hormonal immunosuppressive drugs; CQ+GC: chloroquine combined with glucocorticoids; TCM+GC: traditional Chinese medicine combined with glucocorticoids; TCM+ID: traditional Chinese medicine combined with non-hormonal immunosuppressive drugs.

**Fig 4:**
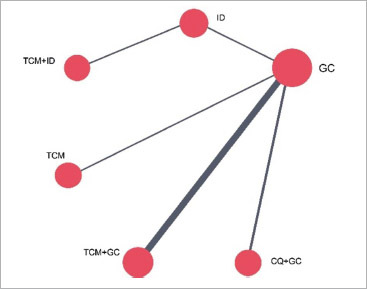
Network evidence plot for side effects. GC: glucocorticoids; PDT: photodynamic therapy; TCM: traditional Chinese medicine, ID: non-hormonal immunosuppressive drugs; CQ+GC: chloroquine combined with glucocorticoids; TCM+GC: traditional Chinese medicine combined with glucocorticoids; TCM+ID: traditional Chinese medicine combined with non-hormonal immunosuppressive drugs.

We then use the R Gemtc package to construct network evidence diagrams for interventions. As illustrated in Figures 3 and 4, each node within the diagram represents a distinct treatment, while the thickness of the lines between nodes illustrates the number of studies that provide the comparison results of the given treatments. Notably, the thickest line is observed between TCM+GC and GC, indicating a higher number of studies comparing their efficacy. Conversely, a thinner line is found between CQ+GC and GC, suggesting fewer comparative studies. The presence of closed loops in the diagram prompts the need for consistency checks. Regarding the 10 studies that reported side effects, it was observed that six types of treatment, such as TCM, TCM+ID, TCM+GC, CQ+GC, ID, and GC were possible causes, while there is no case caused by PDT.

### NMA Analysis Results

#### VAS score

**Fig 5:**
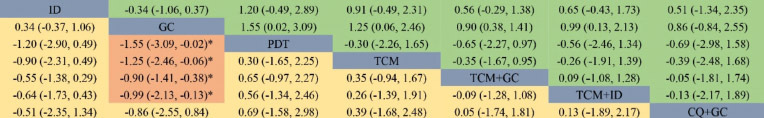
Relative effects in VAS score of multiple treatments. GC: glucocorticoids; PDT: photodynamic therapy; TCM: traditional Chinese medicine, ID: non-hormonal immunosuppressive drugs; CQ+GC: chloroquine combined with glucocorticoids; TCM+GC: traditional Chinese medicine combined with glucocorticoids; TCM+ID: traditional Chinese medicine combined with non-hormonal immunosuppressive drugs.

**Fig 6:**
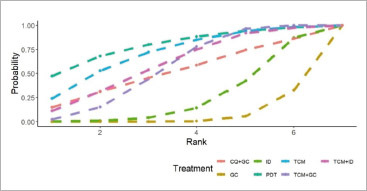
Cumulative probability ranking chart of VAS score. GC: glucocorticoids; PDT: photodynamic therapy; TCM: traditional Chinese medicine, ID: non-hormonal immunosuppressive drugs; CQ+GC: chloroquine combined with glucocorticoids; TCM+GC: traditional Chinese medicine combined with glucocorticoids; TCM+ID: traditional Chinese medicine combined with non-hormonal immunosuppressive drugs.

After conducting heterogeneity tests on VAS score data using the R Gemtc package, it was found that the heterogeneity index I^2^ was 6%, indicating strong goodness of fit and good consistency in the results. Consequently, a consistency model was employed for NMA analysis. Figure 5 illustrates that four treatments demonstrate a significant, in statistic, decrease in VAS score, compared to GC, and PDT has revealed the largest scale (–1.55, 95% CI: (–3.09, –0.02)), and followed by TCM (–1.25, 95% CI: (–2.46, –0.06)), TCM+ID (–0.99, 95% CI: (–2.13, –0.13)), and TCM+GC (–0.90, 95% CI: (–1.41, –0.38)). Moreover, based on the probability of SUCRA probability, depicted in Figure 6, the ranking of our interested treatment is as follows: PDT > TCM > TCM+ID > TCM+GC > CQ+GC > ID > GC. According to the findings above, it is reasonable to conclude the treatments PDT, TCM, TCM+ID, and TCM+GC have greater efficacy than the traditional treatment GC, especially PDT from the perspective of VAS score decrease.

### Results of Side Effects

**Fig 7:**
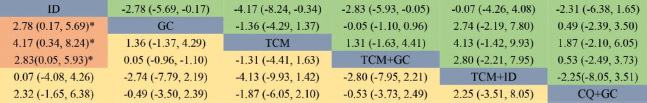
Side effects of multiple treatments. GC: glucocorticoids; PDT: photodynamic therapy; TCM: traditional Chinese medicine, ID: non-hormonal immunosuppressive drugs; CQ+GC: chloroquine combined with glucocorticoids; TCM+GC: traditional Chinese medicine combined with glucocorticoids; TCM+ID: traditional Chinese medicine combined with non-hormonal immunosuppressive drugs.

**Fig 8:**
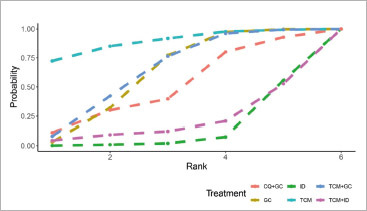
Cumulative probability ranking chart of side effects. GC: glucocorticoids; PDT: photodynamic therapy; TCM: traditional Chinese medicine, ID: non-hormonal immunosuppressive drugs; CQ+GC: chloroquine combined with glucocorticoids; TCM+GC: traditional Chinese medicine combined with glucocorticoids; TCM+ID: traditional Chinese medicine combined with non-hormonal immunosuppressive drugs.

Among the 20 studies included, a total of 10 reported 97 cases of side effects, 5.81% of the entire sample, all categorised as mild adverse reactions such as dizziness, nausea, and decreased appetite. Consistency was deemed superior upon comparison of consistency and inconsistency models, with a heterogeneity test yielding a result of I^2^ = 10%, indicating a well-fitting model. Consequently, a consistency model was chosen for the NMA analysis. Figure 7 elucidates that three treatments demonstrate a statistically significant decrease in the odd ratios, compared to ID. The treatments, in descending order of the odd ratio, are TCM (–4.17, 95% CI (–8.24, –0.34)), TCM+GC (–2.83, 95% CI (–5.93, –0.05)), and GC (–2.78, 95% CI (–5.69, –0.17)). According to the probability of SUCRA probability, depicted in Figure 8, the ranking of our interested treatments is as follows: TCM > TCM+GC > GC > CQ+GC > TCM+ID > ID. In addition, from our studies, there is no side effects case caused by PDT treatment. These findings indicate that among the six treatments, TCM, TCM+GC, and GC are significantly less likely to cause side effects, compared to ID.

### Publication Bias

**Fig 9:**
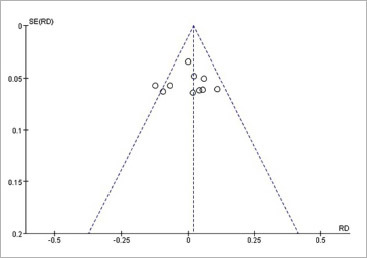
Funnel plot of VAS score.

**Fig 10:**
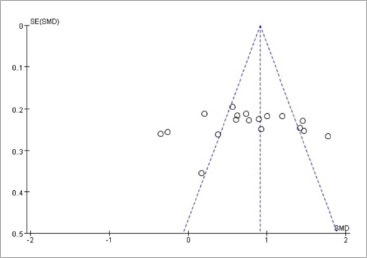
Funnel plot of side effects.

Funnel plots were created using RevMan 5.4.1 software to depict the distribution of VAS scores and side effects. The symmetric distribution of the plots, shown in Figure 9 and Figure 10, suggests minimal publication bias in the present study.

## DISCUSSION

OLP is a common disease affecting the oral mucosa, characterised by unclear pathogenesis and carcinogenic mechanisms. Despite the lack of definitive therapeutic approaches, GC such as triamcinolone acetonide, dexamethasone, prednisolone, and mometasone furoate remain the frontline medications for treating OLP in clinical practice. This is attributed to their ability to suppress the body’s immune function, alleviate allergic symptoms, and inhibit the secretion of inflammatory mediators. However, besides Western medicine, the value of TCM began to be recognised in recent decades and has been accepted as a potential candidate for alternative therapy in OLP.

Therefore, researchers dedicated themselves to comparing the efficacies between Chinese and Western medicine in recent years. This research selected 20 studies, a total of 1669 samples, and compared seven commonly used treatment approaches for OLP. Our results show that PDT and TCM, as well as TCM combined with GC and ID, are more effective treatments compared to using GC alone, with statistically significant results. PDT is particularly advantageous due to its minimal side effects, making it a unique non-pharmacological treatment option. TCM alone causes fewer side effects than ID, and TCM combined with GC is statistically better than using ID or GC alone, offering protection against GC-induced side effects. These findings suggest that PDT and TCM could be promising alternative treatments for OLP in the future.

TCM is an ancient medical practice originating from China with a history of over 2000 years. Initially, it could be considered a medical system based on experience. However, over a long period of development, it has formed an independent and comprehensive theoretical framework. Taking OLP as an instance, TCM theories posit that OLP results from functional imbalances in Xin, Gan, and Shen, leading to obstruction in energy flow. This imbalance is often termed ‘Xu huo’, resembling inflammation in Western medicine. It arises from a disruption in the body’s yin and yang balance. This abnormal state manifests on the surface of the oral cavity and other body areas.9 To mitigate symptoms, TCM often utilises approaches like heat-clearing, inflammation reduction, and liver and kidney nourishment. Many studies agree that TCM interventions can effectively alleviate the symptoms of OLP.^5,29^

Even though TCM presents superior efficacy in treating OLP, many physicians outside China only recognise the potential therapeutic value of specific individual traditional herbal ingredients in treating oral diseases like OLP. The lack of knowledge of the TCM theoretical framework brings a restriction in the development and popularisation of TCM.^39^ Therefore, we look forward to encouraging clinical physicians to understand the TCM theories by verifying the value of TCM.

In addition, it is noteworthy that research has identified intense and sustained negative emotions as one of the risk factors for OLP.^11^ Negative emotions, including anxiety, stress, and sadness, are known to be common triggers for OLP and can worsen symptoms in affected individuals.^23^ Consequently, comforting patients and reducing their negative emotions, while simultaneously enhancing their sense of well-being are viewed as potential strategies to assist in the treatment of OLP.^1^ Our research did not include the consideration of the features of the patient’s mental and emotional states, which could be the orientation for future research. Notably, recent studies have demonstrated that PDT exhibits remarkable efficacy and is devoid of side effects. These findings underscore the promising application of PDT in managing OLP, suggesting a safe and effective therapeutic approach for this condition.^30^ Recent research has suggested a correlation between the efficacy of PDT and the type of laser used, with semiconductor lasers potentially showing superior results in reducing VAS scores compared with diode lasers.^14^ Furthermore, studies have shown that different combinations of TCM ingredients and the combination of TCM with PDT may have varying therapeutic effects on OLP.^12^

## CONCLUSION

This paper, through an NMA, found that PDT and TCM are superior methods in treating OLP than conventional treatments. Moreover, TCM combined therapies, such as TCM + ID and TCM + GC, perform a lower likelihood of side effects happening than the treatment using GC alone. Future research should use standardised clinical trial data and consider patient emotions, lifestyle, and treatment duration to better understand TCM’s effectiveness in treating OLP. Comparing different TCM combinations and their synergy with conventional treatments will help identify more effective therapies. This can expand TCM’s role in oral mucosal disease treatment, encourage its clinical use, improve patient experiences, and enhance efficacy.
